# Views of German mental health professionals on the use of digital mental health interventions for eating disorders: a qualitative interview study

**DOI:** 10.1186/s40337-024-00978-1

**Published:** 2024-02-23

**Authors:** Gwendolyn Mayer, Diana Lemmer, Ina Michelsen, Pauline Schrader, Hans-Christoph Friederich, Stephanie Bauer

**Affiliations:** 1 Department of General Internal Medicine and Psychosomatics, Heidelberg University Hospital, Im Neuenheimer Feld 410, 69120 Heidelberg, Germany; 2 Center for Psychotherapy Research, Heidelberg University Hospital, Bergheimer Str. 54, 69115 Heidelberg, Germany; 3 German Center for Mental Health (DZPG), partner site Mannheim/Heidelberg/Ulm, Germany

**Keywords:** Mental health care, Eating disorders, Qualitative research, Implementation barriers, Implementation facilitators, Views, Opinions, Risks, Digital mental health

## Abstract

**Introduction:**

Digital mental health interventions (DMHIs) are getting increasingly important for mental health care. In the case of eating disorders (EDs), DMHIs are still in early stages. Few studies so far investigated the views of mental health professionals for EDs on the integration of DMHIs in routine care.

**Objective:**

To gain insights into the experiences, perspectives, and expectations of mental health professionals for EDs regarding DMHIs and to identify requirements for the future integration of DMHIs into routine care.

**Methods:**

Semi-structured qualitative telephone interviews with 24 German mental health professionals treating patients with EDs were conducted. A content analysis following a deductive-inductive approach asked for experiences, advantages and chances, disadvantages and boundaries, desired functions and properties, target groups, and general conditions and requirements for DMHIs for patients with EDs.

**Results:**

Only few professionals reported experiences with DMHIs besides video-based psychotherapy during the pandemic. From the therapists’ point of view, DMHIs have the potential to deliver low-threshold access for patients with EDs. Useful functionalities were seen in digital meal records, skills training, and psychoeducation. However, a stable therapeutic alliance was reported as an important prerequisite for the successful integration into care. Therapists expressed concerns in case of severe anorexia nervosa or suicidality. The participants felt to be informed inadequately on recent developments and on the evidence base of DMHIs.

**Conclusions:**

Mental health professionals for EDs show positive attitudes towards DMHIs, however many barriers to the integration in routine care were observed. The highest potential was seen for the use of DMHIs in addition to outpatient care and in aftercare. Specific requirements for DMHIs are related to different areas of the healthcare spectrum and for the different symptom profiles in anorexia nervosa, bulimia nervosa and binge eating disorder. Targeted DMHIs are needed and appropriate especially for concepts of blended care.

**Supplementary Information:**

The online version contains supplementary material available at 10.1186/s40337-024-00978-1.

## Abstract (plain language summary)

Digital mental health interventions are therapeutic services for people with a mental disorder that can be delivered on electronic devices. They are getting increasingly important, as many patients have to wait long for a therapy. In eating disorders these interventions are still in early stages. Twenty-four telephone interviews were held with German professionals treating adolescent and adult patients with eating disorders. The aim was to understand their experiences, perspectives, and expectations regarding digital mental health interventions and to find out what is needed to integrate them into care in the future. In general, the interview partners showed positive attitudes towards these interventions. However, only few reported experiences and many obstacles were observed. The highest potential was seen for the use in addition to care outside the hospital setting and after treatment has ended. However, in case of a severe eating disorder, such as anorexia, or self-harm and suicidality, they were against the use of digital interventions. A stable personal relationship to their patients was seen as particularly important before recommending a digital intervention. Finally, the interview partners felt not informed in a sufficient way on the scientific basis and regulations regarding digital mental health interventions.

**Supplementary Information:**

The online version contains supplementary material available at 10.1186/s40337-024-00978-1.

## Introduction

### Background

Eating disorders (EDs) cover a number of serious mental disorders in their three main forms: anorexia nervosa (AN), bulimia nervosa (BN), and binge eating disorder (BED). Their lifetime prevalence in Western countries is estimated to be 1.9% for any ED in both sexes and up to 2.6% for women [1]. Patients suffering from an ED have a substantially reduced quality of life due to a high disease burden, a chronic disease progression, mental and physical comorbidities, and an increased mortality rate [2]. Typically, EDs show an age of onset in early adolescence with a peak incidence period of 13–18 years [3]. However, an increasing number of children are also affected and often remain untreated [4]. There is still a huge gap between treatment needs and the timely provision of support due to a lack of therapists on the one hand and patient-related factors on the other. Many patients still face stigma and shame, or fail to see the severity of their illness [5]. A recent analysis of secondary data from Germany has shown that only a quarter of those affected by an ED receive outpatient psychotherapy [6].

Electronic devices get increasingly important for the delivery of support. Especially during the pandemic, a substantial share of patients with an ED received psychotherapy with the help of a video-based software [7]. But even apart from contact restrictions during the pandemic, so-called digital mental-health interventions (DMHIs), also known as e-health, internet-based, web-based, or online interventions, got more and more present as an emerging field of innovative delivery of mental healthcare. They can include many components, such as mood tracking or cognitive tasks and may be delivered on different devices, such as mobile phones, tablets, or computers. An increasing offer of DMHIs for the most common disorders, such as depression and anxiety, is available in app stores with lacking scientific evidence [8, 9]. However, growing evidence has been shown in randomized controlled designs of DMHIs that follow a guided cognitive behavioral therapy format, i.e. offer therapeutic support [10, 11].

Still, the evidence regarding DMHIs for EDs remains limited. A review of Ahmadiankalati et al. [12] identified 12 RCTs with a variety of interventions with and without therapist support. Linardon et al. [13] reviewed 36 RCTs on DMHIs for EDs, but only 8 of them targeted treatment. Both reviews concluded that the studies suffer from low quality so far, high drop-out rates, and inconsistent intervention acceptability. Yet, there is a growing number of smartphone apps in app stores related to EDs. Two recent reviews investigated the types and therapeutic components of apps available in the marketplace. First, Wasil et al. selected 28 apps and found some elements of empirically supported treatments such as self-assessments, cognitive restructuring, and activity scheduling. However, only four apps were used frequently, and the authors recommend clinicians to get familiar with these interventions as patients might have had experiences with them [14]. Additionally, another study team found 65 apps in 2021, and only seven percent of them had been scientifically evaluated. Again, most of the literature focused on a small number of apps [15].

Specifically, adolescents show a high vulnerability for EDs, which has even increased during the pandemic [16]. There is only a limited number of studies investigating the effectiveness of DMHIs for the age group until 18, a review on 4 studies showed a decrease in ED symptomatology [17]. A more recent scoping review with a broader age group as target participants (10–25) assessed 49 studies that showed moderate to large effects in symptom reduction for video-based psychotherapy, but inconsistent results for internet self-help programs and no effects for mobile applications [18]. Only few studies investigated the use of mobile applications, that were perceived as acceptable but lacking privacy and features of personalization. Small to moderate effects were observed for mobile apps, if they were used within a hybrid format adjunctive to an ongoing face-to-face therapy [18].

An important prerequisite for the effective use of DMHIs is their acceptance. Individuals in Australia, the U.S. and the United Kingdom with self-reported symptoms but no diagnosis have been found to be more positive about internet-delivered or mobile interventions than individuals who have been diagnosed with an eating disorder [19]. To improve patients’ access to DMHIs, clinicians have been identified as potential gatekeepers. This shows the importance of understanding concerns that they may raise and obstacles that they may see. Main issues identified in the past related to financial and regulatory questions, such as reimbursement, credentialing and liability [20]. In Germany, the recently launched digital healthcare act provided legal regulations for the clinical use of certified digital interventions [21]. A previous study of the first author few months before the launch of this act has shown that medical experts in Germany had only little knowledge of DMHIs [22]. Another German study team found that even two years after the new legislation, the uptake and usage of these interventions are still slow and healthcare providers are reluctant to prescribe DMHIs [23].

To date, little is known about the views of clinical experts for EDs on the application of DMHIs. Only one study so far has put a focus on the perspectives of German stakeholders regarding online interventions for EDs [24]. In this study, stakeholders were defined as either potential users (patients and caregivers), decision-makers (e.g. health authorities), or facilitators (mental health care professionals, including social workers and nurses). The third group took part in an online survey. The results showed that only 14.6% of the professionals had personal experiences with DMHIs for EDs, less than half had at least looked into such an application, and nearly 30% had never heard of them. However, in-depth insights on the risks and implementation barriers of DMHIs for EDs as expressed by mental health professionals are missing so far, and insights on expectations and potential advantages for ED healthcare are scarce.

### Objectives

This study aims to understand the experiences, perspectives, and expectations of mental health professionals for EDs regarding DMHIs. To what extent do they already use DMHIs in their treatment? Which advantages and chances do they see in their usage? What disadvantages and risks do they perceive? A final focus will be put on specific requirements for the potential integration of digital applications into routine care.

## Methods

### Study type

This study used a qualitative research design to investigate the experiences, perspectives, and expectations of mental health professionals for EDs towards DMHIs. Ethical approval was obtained by the Ethics Commission of the Medical Faculty at University of Heidelberg (S-178/2022).

### Recruitment and procedures

We invited clinical experts for EDs throughout Germany from inpatient clinics, outpatient clinics, and private practices to take part in a 30–60-min semi-structured telephone interview. Invitations were sent out via e-mail to practitioners with an expertise in the field of EDs. Potential participants were identified both conveniently (i.e. contacts of the authors who fit into the inclusion criteria) and purposively through an internet search process. All participants received written information on the aims and procedures of the study. Informed consent was given electronically and confirmed on the telephone prior to the interviews. The interview guide included questions about previous experience with digital services or programs, advantages and disadvantages, framework conditions and prerequisites, as well as expectations towards an ideal DMHI for EDs. At the beginning of the interviews we explained what kinds of technology might be included in the term DMHI and provided a list with non-exhaustive examples (e.g. online counseling by email or chat; video conferencing; mobile apps; fitness bracelets; online programs; virtual reality). The two interview guides for clinicians in the treatment of children/adolescents and adults are included in Additional files 1 and 2. Participants received gift vouchers worth 100 Euros for their participation in the qualitative interviews and a subsequent quantitative study. Interviews were conducted between April and July 2022 at the Center for Psychotherapy Research, Heidelberg after interviewer trainings.

### Sample description

A total of 24 mental health professionals were included in the study. The age of the participants ranged from 26 to 58 years (*M* = 41.96, *SD* = 9.92). Further demographic details are shown in Table 1.

**Table 1:** Demographic characteristics

Characteristic	Category	Frequency (percentage) n (%)
Gender		
	male	2 (8.3)
	female	22 (91.7)
Type of institution		
	hospital	17 (70.8)
	outpatient services	1 (4.2)
	residency	3 (12.5)
	mixed	3 (12.5)
Professional background		
	psychology	14 (58.3)
	medicine	7 (29.2)
	pedagogy	2 (8.3)
	social work	1 (4.2)
Status		
	with approbation	21 (87.5)
	still in professional training	3 (12.5)
Clinical orientation (multiple categories possible)		
	cognitive behavioral therapy	18 (75.0)
	psychodynamic therapy	5 (20.8)
	systemic therapy	3 (12.5)
Patient group		
	adults	13 (54.2)
	children & adolescents	8 (33.3)
	both	3 (12.5)
Total		24 (100)

### Data analysis

The duration of the interviews was between 35 and 60 min (*M* = 50′, *SD* = 07′). The audio recordings were transcribed verbatim and analyzed by two coders, who were both trained psychologists (GM, DL). The analysis was carried out in MAXQDA [25].

The analysis followed the rationale of a deductive-inductive content analysis, which combines best practices from two coding techniques [26] and has as well been indicated as an abductive or complementary approach [27]. The majority of codes were generated from the material, which refers to inductive coding [28]. However, some questions directly asked for certain aspects, such as "advantages" or "risks". In these cases, the codes were assigned according to the interview guide. After creating initial codes, all codes were compared and assigned to a coding structure of main codes and subcodes. The two coders, who were both trained psychologists, compared their results in several iterations, refined the coding structure, and agreed on a joint definition in an iterative process. As soon as all codes and subcodes covered the meaning of the data, the decision was made on thematic saturation.

Due to the complexity of the topic, single expressions of the interview partners could be coded with multiple codes and subcodes. By this, nine main codes and subcodes with two levels were assigned to statements of the interview partners (Table 2). In the results section details of the codes 4–9 are elaborated. All main codes including the three others, all subcodes, and examples for supporting quotes are provided in Additional file 3.

**Table 2:** Coding system with main codes and first level subcodes

Main code	Subcodes
(1) Initial situation of the interviewed person	working conditions, treatment setting; treatment role of other professional and informal caregivers/ personal contacts and other experts; sources of information; patient characteristics; general media use; provision of care
(2) General attitudes towards DMHIs	curiosity, interest; skepticism, insecurity; future prospects
(3) General societal conditions	care supply in general; social media; COVID-19 pandemic
(4) Experiences with DMHIs	own involvement in DMHIs developments or evaluations; impact on therapeutic process; specific functionalities; media; patient characteristics; experiences of patients/patient perspective & feedback; setting
(5) Advantages and chances	advantages for relatives/informal caregivers; advantages for service provision; advantages for patients
(6) Disadvantages and boundaries	disadvantages for service provision; disadvantages for patients
(7) Desired functions and properties	design; medium; technical functionalities; therapeutic content
(8) Target groups of DMHIs	patients’ age; motivation, initiative, interest; media competency; diagnosis; gender; contraindications; social environment, relatives
(9) General conditions and requirements	spatial conditions; scientific evidence; costs, finances; training; indication; health insurance; frequency and degree of use; availability of DMHIs; staff prerequisites; legal conditions; therapeutic setting; technical requirements; therapeutic alliance as a basis

## Results

### Experiences with DMHIs

Even though all participants reported to have some experience with DMHIs, the interviews showed that these referred in most cases to video-based systems during or after the pandemic. Especially therapists for children and adolescents appreciated the opportunity to have a cost-effective way to get in touch with parents in remote areas. Therapists for adults also assessed their experiences as beneficial. However, many interview partners expressed that video-based psychotherapy will never replace a face-to-face therapy. Negative experiences related to patients with a high disease burden and potential suicidality:

‘*…and with the video-based system, I've also had patients who really had a suicidal crisis, who started hurting themselves again, and I would have preferred it if we could have discussed this face-to-face and not through a video-based format*’ (psychological psychotherapist, adult patients, inpatient care, female, 26 years)

Very few professionals reported experiences with specialized DMHIs for EDs. Some of those knew the smartphone application “Recovery Record” [29]*,* others mentioned experiences with digital meal records without naming the original title of the app. The experiences with this functionality was perceived as a useful treatment adjunction, as expressed by a therapist for adult patients:

‘*Well, I think I have a pretty good therapeutic relationship with most of the patients. But of course, I've already noticed, like now with this one patient, who is now continuing the meal log, the impression that it gives a lot of security and tends to strengthen the relationship. Yes, now over these eight weeks that she is now inpatient somewhere else*’ (medical doctor, inpatient care and day-care, adult patients, female, 37 years).

Other positive experiences referred to apps providing relaxation or awareness trainings. Skills trainings and psychoeducation were mainly used for patients with obesity. Some participants talked about participating in current studies and made positive statements, e.g. regarding the use of virtual reality for confrontation exercises or training units focusing at the body image.

### Advantages and chances

Advantages of DMHIs were categorized in those for patients and those for healthcare provision. Additionally, a few advantages for relatives were mentioned. As a direct advantage for patients, many participants appreciated DMHIs to be an easily accessible, low-threshold way for patients from remote areas or without medical treatment to a first contact with mental healthcare services. Moreover, as many patients feel shame or experience fear of stigma, a DMHI might be a first step for them to access support or ED-related healthcare. In this context, psychoeducation provided in a digital form was seen as beneficial.

Mental health professionals for EDs for children and adolescents observed that many young people spend a lot of time with their mobile devices anyway and could be met by DMHIs where they already are, i.e. in the digital space. Therefore, DMHIs were seen to be close to daily life.

Another aspect mentioned by the participants was that DMHIs might increase treatment adherence, if it targets the individual needs of a patient. This was expressed by a therapist for adults who said:

‘*I could imagine that this aspect of being taken seriously, that this could actually benefit from digital interventions, because what actually happens from time to time, especially when the patients are not very young, when you give them a worksheet or something that sometimes makes them feel like they are in school. So now there is homework, so to say, and I could imagine if you had such an interactive digital tool, and they could do that explained and so that could be a bit catchier.*’ (psychological psychotherapist, outpatient care, adult patients, female, 31 years)

However, other advantages were seen in quality improvements of healthcare by the delivery of DMHIs. As EDs in general are not easy to treat, and some patients do not benefit from psychotherapy, DMHIs could enhance treatment. Beyond that, many patients are in urgent need for psychotherapy but still need to wait several months for treatment. Evidence-based DMHIs for the treatment of EDs would help to reduce this gap:

‘*In general, I would say, first of all that it offers the opportunity to provide much, i.e. better, care. We are now seeing in the aftermath or even during the pandemic that the need has increased enormously, the patients are also significantly sicker, and we cannot respond with a corresponding offer, or even on the contrary, due to illnesses of colleagues and also pandemic-related challenges for the clinics, sometimes the space available is even less, even smaller. And of course, there the use of digital media is a great help in reaching the patients.*’ (Psychological psychotherapist, inpatient care, adolescent patients, female, 41 years)

### Disadvantages and boundaries

All participants were aware of risks and limitations of DMHIs for EDs that were grouped as disadvantages for patients and those for healthcare provision. Disadvantages for patients related to issues of data security, lacking personalization, or the fact that DMHIs might not be an adequate and sufficient help. In some cases, the participants even perceived a danger that the disorder itself might worsen.

‘*I would be concerned that it would be too much about, … and then turning in an unhealthy direction, like 'how many calories do I burn' and 'how much do I move' and 'how much do I weigh'*. *So that's it. Yes, it's also painful for many patients, if it's just about that, I wouldn't expect that from the app, but I don't think that would be good if it's just about weight*.’ (psychiatrist for children & adolescents, inpatient care, adolescent patients, female, 38 years)

A major threat for the quality of treatment was seen in the potential impairment of the therapeutic alliance that might occur with DMHIs. On the one hand, concerns were raised that patients might use the DMHI as an insufficient replacement for a face-to-face psychotherapy due to its convenience and comparatively low effort. On the other hand, the physical presence in treatment was seen as a prerequisite for change during therapy. One therapist saw this as crucial for the treatment of patients with AN:

‘*… regular weighing is not something that we completely outsource to the paediatricians, but we weigh them here as well, because weighing is also important for exposure, so that they learn to bear the higher number on the scale. And I think that's something that works better when you're close to it.*’ (Psychological psychotherapist, outpatient care, adult and adolescent patients, female, 47 years)

Furthermore, participants saw the risk that in case of a suicidal crisis or self-harm, the clinician in charge might be informed too late to intervene in time. Even in video-based therapy, this was seen as a major risk, since only parts of the patient are visible for the therapist, not the whole body. Nonverbal communication was regarded as an important element to assess the health status of the patient.

### Desired functions and properties

When asked for an ideal DMHI for their patients, all participants expressed their ideas and wishes. These desired functions and properties were grouped into those related to the design of the DMHI, the respective medium (e.g. app, pc), technical functionalities and to the therapeutic content.

The majority of properties wished by the participants aimed at functionalities for mobile applications rather than other devices. In the first line the participants talked about mobile apps for symptom and treatment monitoring. Depending on the respective diagnosis this could be a mood tracking functionality or a meal protocol, that has to be filled out either retrospectively or for future planning. Retrospective protocols were seen as beneficial for analyzing critical situations, e.g. for patients with binge eating episodes. Meal plans were favorized for patients with AN. Other functionalities referred to psychoeducational content or skills training, that could be activated at a specific time point in therapy as suggested by one participant:

‘*Of course, it would be good if the therapist could also simply activate specific topics over the course of the process, so I think that would also be very nice if you noticed: Okay, now self-esteem is somehow a big topic for the patient, then you get in, then you have the option as a therapist to unlock the self-esteem block for the patient, something like that, that would be really cool, well.*’ (Psychological Psychotherapist, outpatient care, adult patients, female, 40 years)

A critical point was the question if the application should allow access for therapists or even relatives. Participants in favor of the opportunity to give therapists access to the content patients had worked on, argued that this would be beneficial for keeping contact with the patients and for sending reminders. However, one therapist insisted that patients should not be able to see when exactly she is available and if a message has already been read, as opposed to text messaging programs. Other mental health professionals appreciated the opportunity to stay in contact with a patient after treatment as a way of digital after-care.

Clinical experts who argued against a shared access said that patients might feel observed and so would not really work on their problems while using the intervention:

‘*I also believe that in case of doubt, depending on the … status in therapy, this changes the benefit and maybe also prevents it, because such a social desirability has a great influence. Well, I think as a patient, if I put my mind to it, I might have situations in which I wouldn't use it, because it would be so uncomfortable that my therapist would see it afterwards.*’ (Psychosomatic medicine (in qualification), day-care, adult patients, female, 40 years)

Most participants argued against an access for relatives to the DMHI of a patient. Exceptions were made in case of video-based therapy, where it was perceived as useful to meet parents at different time points of a psychotherapy. Moreover, separate tools or virtual groups only for relatives of a patient with ED were suggested.

### Target groups of DMHIs

The interview partners talked about specific characteristics of the respective target groups of DMHIs in an elaborate and detailed way. In general, a prevalent view was that there would never be a “one-size-fits-all” digital solution for individuals affected by EDs. Some functionalities might be supportive for all kinds of EDs, such as meal records, meal planning, skills training, and planning of activities. But the adequate assignment of ED specific tools that might be available in the future should depend on the age of the patients, the motivation to change, and to a certain extent on the exact diagnosis and comorbidities. Activity planning, for example, might include suggestions for sports and motion for patients with BN and BED, who have to overcome their fears of sports. Patients with AN, in turn, should rather be encouraged to reduce their urge to exercise and do workouts excessively.

Some functionalities could be very helpful for patients with AN, such as assessing the size of meals:

‘*So … what comes to me spontaneously now … patients with anorexia … they often have difficulties when it comes to estimating portion sizes and if there was such an app now, it would take a photo, so to speak, with the camera from your cell phone, if you hold it on the plate and then somehow could compare what would have been, what they should have eaten and what, just then, wouldn't have been.*’ (medical doctor, inpatient and outpatient care, adult patients, female, 43 years)

However, the usefulness of DMHIs for patients with AN was assessed to depend on their current body mass index (BMI). Most concerns were raised against incautiously using DMHIs with those affected by severe AN with a low BMI who were characterized as over-controlling in nearly every aspect related to their illness. One medical doctor even saw the use of digital devices as a root cause for the AN of his patient:

‘*I can think of one patient … you could almost say she became anorexic because of her Apple Watch. And that was a huge act in therapy, that this watch more or less put it down and straightened it and buried it. She's sold it now, I think. This ‘come on, you can still do it’, or this: ‘more exercise, and a little fewer calories today’. And this ‘push, push, push’, that was so extreme. So that was really impressive, she developed a massive anorexia when she bought this smartwatch or she was given it as a gift, because everything just turned around the clock, so to speak. So that was, for me too, really impressive to see.*’ (Medical psychotherapist, inpatient and day-care, adolescent patients, male, 55 years)

For BN and BED, the participants saw more positive opportunities, as these patients were reported to often suffer from shame and fear of stigmatization. For them, according to the mental health professionals for EDs, a DMHI might be a very suitable, low-threshold facilitator to help-seeking. Digital monitoring in bulimic patients was assessed as a great way to analyze potential triggers:

‘*Here, I think, it would also be helpful, for example, to have the opportunity to take a closer look at binge eating afterwards, to analyze it, maybe also via an emotion log, to remember a little bit in which situations it would be helpful maybe helpful strategies to use to avoid binge eating, how, what skills can I use, and so on.*’ (Psychological psychotherapist, inpatient care, adolescent patients, female, 41 years)

Clear contraindications for the use of DMHI were seen in suicidality, severe self-harm and problematic media use (i.e., internet or gaming addiction).

### General conditions and requirements

The effective use of DMHIs for the benefit of patients with EDs depends on several general conditions and requirements, as expressed by the interview partners. They observed a broad range of requirements with very different levels of complexity. Many interview partners said that technical requirements, especially in clinical institutions, are often not fully met for the successful integration of DMHIs. As an example, some clinical experts had to use their private mobile phone for a DMHI, even though they would have preferred to be provided with a professional mobile phone. Besides, trained personnel and adequate spaces for their use were mentioned as important conditions. For instance, a separate room for video-based therapy in a multi-person household was viewed as an important prerequisite for patients to ensure privacy.

A major concern focused on data security and privacy, directly followed by a valid legal framework that has to be established, as some professionals feared legal consequences.

‘*From a legal point of view, the issue of data protection is of course a huge issue. So what kind of data do they want to put in there from me, what is done with the data, who gets the data, does the health insurance company get it, is it all stored wonderfully somewhere. So that's a big, big topic. The question of data security and what will happen, millions of data will be generated and what will be done with the data.*’ (Medical psychotherapist, inpatient and day-care, adolescent patients, male, 55 years)

Many interview partners voiced the need for more scientific evidence with specific information regarding treatment mechanisms, indications and contraindications. Nearly all of them felt not to be trained and informed adequately on standards and on the availability of suitable DMHIs.

One main aspect of general conditions and requirements was an appropriate setting for DMHIs. Nearly all participants expressed concerns about DMHIs for unguided self-management. However, as an adjunct to ongoing inpatient or outpatient treatment, the professionals saw many benefits. For example, participants mentioned that inpatient psychotherapy might be accompanied by meal records or daily mood tracking functionalities. Outpatient services could benefit from homework, journal keeping, protocols, and modules for psychoeducation. Moreover, single in-person sessions could be replaced by online sessions, as long as a good therapeutic alliance has been established before.

Finally, many participants saw benefits in the delivery of a digital aftercare tool in order to stay in contact to patients and build on therapy successes. One expert gave an example:

‘*Then actually after the inpatient stay, … seeing something to prevent relapse in the sense that they might have a kind of traffic light system, am I still running in the green, is it running in the yellow area, I'm already in the red area. As far as symptom behavior is concerned, I could well imagine that too. So how's it going with eating, exercise, decrease in vomiting if that's an issue now, or binge eating.*’ (Psychological psychotherapist, outpatient and day-care, adult patients, female, 55 years)

## Discussion

This qualitative interview study asked mental health professionals for their experiences, perspectives, and expectations regarding DMHIs for EDs. In general, our results show that the ED professionals voice open-minded but critical attitudes on the integration of DMHIs for their patients. This general attitude was independent of professionals’ training in either working with children and adolescents or adults. However, only few clinical experts reported having prior experiences with DMHIs during the pandemic aside from video-based therapy.

All professionals talked about various advantages and disadvantages. Moreover, they were asked about useful technologies and functionalities, they would benefit from if they were available. In the following two sections we give an overview on specific requirements of the future integration of DMHIs in routine care in the context of the current literature. Our considerations first relate to different areas of the healthcare spectrum and are then grouped by diagnosis.

### Requirements for the integration of DMHIs into different areas of the healthcare spectrum

DMHIs for EDs may potentially be used across all areas from prevention, self-management, and treatment to aftercare. Mental health professionals in our interviews saw the greatest potential in the delivery of digitally-enhanced outpatient care, i.e. DMHIs as adjunct to conventional psychotherapy. In this context, the most important aspect was that DMHIs were assumed to be useful as soon as a positive therapeutic alliance has been established. In fact, the alliance has been shown to be an important factor in internet-based interventions for mental disorders in a meta-analysis of 18 studies [30]. Only few studies investigated the role of the alliance in DMHIs specific for EDs. In the Dutch program “Look at your eating” (Etendebaas) the therapeutic alliance was predictive for pathology and treatment completion [31]. However, psychotherapy research has also shown that the relationship between the therapeutic alliance and treatment outcome is bidirectional, i.e. early symptom improvement predicts subsequent improvements in the alliance and vice versa [32]. In line with this, the alliance with the therapist accompanied by the confidence in the own ability to change can be improved by the supportive use of DMHIs in addition to outpatient care [33]. Outpatient care, in turn, plays a major role in the promotion of access to DMHIs. A recent study of Dahlhausen et al. showed that especially clinicians in outpatient settings are more able to promote adherence to DMHIs than those in hospitals, because of the long-lasting relationships they have with their patients [23]. To sum up, clinicians see the therapeutic alliance as a facilitator for the integration of DMHIs into routine care and results from the literature support this assessment. This is as well supported by the observation that the clinicians are the gatekeepers of patients’ access to DMHIs [20]. When asking for the perspective of individuals with EDs or ED symptoms, studies asking directly for their opinion regarding the alliance are missing so far. However, a randomized controlled study showed that increased therapist support increases satisfaction of individuals with ED symptoms but not symptom improvement [34]. However, users clearly prefer clinician support in DMHIs [35].

Unguided self-management applications were rated critically by the participants of our study. However, the interview partners saw potential of DMHIs in bridging waiting times, as already reported in previous studies [24]. Skeptical voices added that there might be disappointments especially in young people and their relatives if DMHIs would not lead to an immediate improvement of symptoms. There could be the risk that these people might give up and miss a timely intervention.

Evidence on self-help interventions has been established mostly for mental disorders other than ED, e.g. in the case of depression [36], anxiety [37], and obsessive–compulsive disorder [38] usually by comparing a DMHI to a wait-list or usual care, which is not necessarily an evidence-based face-to-face psychotherapy. While many studies have confirmed the potential of self-management programs for ED prevention (i.e. in at-risk samples) or in self-selected samples with no diagnostic procedures, the body of literature on high-quality RCTs providing evidence on self-management interventions for individuals with diagnosed ED is still small [12, 13, 39]. Although patient engagement was high in a the case of a platform for BED [40], internet-based self-help was inferior to face-to-face but still effective for BED [41], and online CBT-based self-help showed good clinical outcomes for patients with BN [42]. There is evidence that such DMHIs are better than no intervention (e.g. when compared to waitlist groups), but not superior to other active conditions (e.g. when compared to bibliotherapy [43]) and inferior to conventional psychotherapy [41]. Recommendations concerning DMHIs should therefore take the respective context into account and their use may be well-justified in cases where no timely conventional treatment is available.

DMHIs for EDs can as well be a useful adjunct for day-care, where patients stay in the hospital during the day, but go home for the night. According to the mental health professionals in our study, such partially inpatient treatment for patients with EDs could benefit from mobile monitoring or digital homework. A study on digitally supported daycare for patients with EDs was carried out during the pandemic. Telemedicine with remote sessions was delivered for a small number of adolescent patients with AN. The concept was successful in case there was a supporting family structure [44].

Finally, aftercare was considered a highly promising setting for the integration of DMHIs, as at this point in therapy a certain mental stability of the patient already can be assumed. Nevertheless, an effective strategy for relapse prevention is needed and here, DMHIs show potential. Although the evidence regarding the effectiveness of digital aftercare is limited and heterogeneous, there are positive results for BN [45]. Another study with patients with binge eating attacks tested an aftercare DMHI. They showed that improvements were observed mainly in those patients, who still suffered from their attacks after hospital discharge [46]. In the case of AN, symptom improvements could be shown as well [47], however, more recent results with current technological advancements are needed.

In summary, the main prerequisite for the integration of DMHIs into conventional healthcare is the definition of the most suitable care settings. According to our findings, these are outpatient treatment, daycare, and aftercare. Outpatient treatment, supported by DMHIs can take several forms, of which a blended care concept is an approach that covers both, conventional, face-to-face psychotherapy and internet-based support by a DMHI. Another model is stepped care, that can either follow a stepping-up approach, i.e. by integrating DMHIs in a very early stage of treatment and by this, preventing symptoms, or a stepping-down concept, that means that a conventional treatment is followed by a DMHI, in order to stabilize treatment success [48]. Both concepts depend on the respective clinical setting to a certain extent. Randomized controlled designs on aftercare by DMHIs show improvements, however, they remain statistically not significant regarding the main outcome of ED symptomatology at follow-up [12]. Nevertheless, patients with ED appreciated the opportunity to use a DMHI after discharge and showed high adherence [49]. Stepped care supported by DHMIs in general reached a high level of acceptability by mental health professionals treating patients with EDs [50]. Beyond this, the necessary legal (accountability, data protection), financial (reimbursement), and technical barriers have to be considered (e.g., spatial resources or devices for therapists, who do not want to use their private mobile phones for treatment).

### Requirements of DMHIs for specific types of EDs

Several requirements for DMHIs emerged regarding specific ED diagnoses. A survey with community-based participants from the general public, in parts suffering from symptoms of BN or BED, revealed that the majority preferred a generic e-health program for any kind of ED over a specific one [35]. The mental health professionals in our study had another view on this topic. While they agreed on many common suitable DMHI modules for all EDs, such as psychoeducation, homework, relaxation, and skills training, some specific functionalities were discussed with respect to the specific ED diagnosis.

Especially with regard to the high urge to exercise that can be observed in some AN patients on the one hand, and avoidance of exercise in some BN or BED patients on the other hand, DMHIs should provide personalized suggestions with respect to individual needs. Moreover, even if meal records were favored for any kind of ED, the assessment of meal portions/ sizes by a mobile app were seen as much more relevant for patients with AN. Apart from that, it would be useful for patients with BN or BED to find out potential triggers for a binge eating episode by journal keeping and mood tracking.

The review of Linardon et al. [13] concluded that, based on 8 treatment-focused randomized-controlled trials, the evidence of DMHIs is stronger for BN than BED, and very limited for AN. The authors argue, that this may be due to concerns that the severity of the condition in AN leads therapists to prioritize intensive face-to-face treatment for these patients. The professionals in our study argued in a very similar way against the use of DMHIs in severe cases of AN, as DMHIs might be triggering, and encourage over-controlling eating behavior in patients. Moreover, a very low BMI might have a negative impact on cognitive functioning and concentration. Patients with AN were further seen as the patient group with a higher need for support in comparison to patients with BN and BED, who don’t need the same care frequency than patients with AN. This result corresponds to the known burden of disease and mortality rates in AN [2].

### Strengths and limitations

To the best of our knowledge, this is the first study that conducted detailed qualitative interviews with mental health professionals for EDs for both, children and adolescents, and adult patients in Germany on their attitudes and experiences regarding DMHIs. However, our study is limited to the healthcare system in Germany and comparisons at an international level remain limited. Moreover, DMHIs were defined very broadly in our interviews, as all types of interventions, such as mobile apps, virtual reality interventions, or online programs were included. Due to the qualitative approach, we were not able to ask for specific facilitators, barriers and obstacles for all types of interventions with regard to the respective types of EDs in a systematic way. As a consequence, important factors specific for single types of interventions, e.g. barriers for the implementation of mobile apps for BN patients, implementation of virtual reality for AN patients etc. may have been overseen. As current technology advancements are rapidly developing and by this reaching a high level of specificity, future research designs should focus on single technologies with their specific barriers and facilitators for the whole diagnostic spectrum of EDs.

## Conclusions

Mental health professionals for EDs show positive attitudes towards DMHIs. However, only few of them already had experiences with DMHIs in their daily practice. Main barriers to DMHI integration refer to legal aspects, data protection regulations, and the quality of technical equipment in clinics or outpatient wards. According to our participants, the use of DMHIs has high potential as an adjunct to outpatient treatment or in aftercare, but not as stand-alone self-management interventions, that individuals with EDs would use independent of conventional psychotherapy. Clinical experts saw helpful functionalities for all types of EDs, however, single modules should be tailored to the needs of AN, BN, and BED. Targeted DMHIs for specific diagnoses of AN, BN, and BED are needed and appropriate especially for concepts of blended care.

## Contributions to the literature

- This study presents results of qualitative interviews with mental health experts for eating disorders who were asked on their views on the implementation of digital mental health interventions (DMHIs) for their patients.
- Mental health professionals see potential for the use of DMHIs in outpatient care and in aftercare, but risks in case of severe anorexia and suicidality.
- A stable therapeutic alliance is an important prerequisite for the successful integration of DMHIs into care.
- Many therapists do not feel informed in a sufficient way on evidence base and regulations.

## Reporting standards

The COREQ criteria for reporting qualitative research were followed [51]. The checklist helped to clarify the procedures, analysis, and interpretation.

### Supplementary Information

**Additional file 1. **Interview guidelines for experts in the treatment of children and adolescents with eating disorders.

**Additional file 2. **Interview guide for experts in the treatment of adult patients with eating disorders.

**Additional file 3. **Summary of the main codes in alphabetic order used for qualitative analysis with their subcodes and supporting quotes.

## Data Availability

The datasets used and/or analysed during the current study are available from the corresponding author on reasonable request.

## Abbreviations

AN: Anorexia nervosa
BED: Binge eating disorder
BN: Bulimia nervosa
DMHI: Digital mental health intervention
ED: Eating disorder
