# Supplementary material for: Views of German mental health professionals on the use of digital mental health interventions for eating disorders: a qualitative interview study
Source: J Eat Disord. 2024 Feb 23;12:32. doi: 10.1186/s40337-024-00978-1 (PMC10885453; doi:10.1186/s40337-024-00978-1)
Supplement: Supplementary file 1 — Additional file 1. Interview guidelines for experts in the treatment of children and adolescents with eating disorders. [file 40337_2024_978_MOESM1_ESM.docx]

# Additional file 1: Interview guidelines for experts in the treatment of children and adolescents with eating disorders

General recommendations for using this guide:

- The following guidelines are intended to structure the qualitative interview.
- Both, the order of the questions and their weighting, can be variably adapted to the flow of the conversation over the course of the conversation.
- It's not about repeating the questions exactly, what's important is that the other person understands the question and starts talking.
- Further questions not listed here may also arise from the course of the conversation.

The guide is made up of main questions (gray cells) and possible questions (white cells). The main questions are intended to encourage the other person to talk openly and freely. The questions are asked in addition to generate relevant information that has not yet been mentioned.

In principle, the interview partners should themselves give examples from the area of digital interventions. If there is a need to provide some guidance, interviewers can refer to the list below.

For example, digital interventions may include one or more of the following applications:

- Online advice (email, chat)
- Video conferencing-based systems
- Mobile (smartphone) apps
- Fitness bracelets, wearables
- Therapeutic, browser-based programs, online self-help programs
- Interventions from the area of augmented/virtual reality
- Others (e.g. biofeedback, game consoles, video games)

| 1 | To begin with, can you tell me something about yourself?  How old you are, what your professional background is, and where you are currently based.  How many years have you been treating children and adolescents with eating disorders? |
| --- | --- |
|  | - What is your university background; what subject did you study? - Which psychotherapeutic methods do you use in your work (e.g. psychoanalysis, CBT)? What psychotherapeutic methods were you trained in (psychoanalysis, CBT)? - In what setting do you work with your patients? (e.g. outpatient/inpatient, group/individual treatments)   *If several activities, disciplines, work settings or institutions are reported: Inquire about the focus, e.g.: “Which of the activities you mentioned do you see as your main activity?”, “From what was named, which institutes do you work at mainly?”* |
| 2 | What role do digital applications play in your personal everyday life? |
|  | - Which digital applications do you usually use? On which devices do you use it and how often? - What do you use digital applications for? What tasks or functions do these applications perform? - How would you describe your everyday experience with digital applications? What effects does the use of Internet applications have on your personal everyday life? - What advantages or opportunities do you see in your personal use of digital applications? - What disadvantages or risks do you see in your personal use of digital applications? - *See the list above for suggestions* |
| 3 | How do you rate your personal skills in dealing with digital applications? |
|  | - In which areas do you feel confident when dealing with digital applications? - In which areas do you feel insecure when dealing with digital applications? To what extent do you still see potential in yourself to learn more about it? - Who or what could support you in learning more about handling digital applications? |
| 4 | Could you describe to me the group of children and adolescents you normally deal with in your professional life? (What distinguishes these children and adolescents?) |
|  | - How old are the children and adolescents you work with? - Are they usually more likely to be girls or boys? - What is the setting like in which you work with the children and adolescents? - How do these patients come to you? (Are they referred, recommended, or are they self-initiated?) - Do these children and adolescents come alone or accompanied? If accompanied: who accompanies them? - What role do the caregivers of the children and adolescents play in the treatment? How are relatives, parents or teachers involved in your work, for example? - Which other people are important in your work with children and adolescents (e.g. employees of the youth welfare office)? What role do these people play in the treatment? |
| 5 | What experiences have you already had with digital interventions in your professional practice? |
|  | With which groups of patients did you have these experiences?  (e.g. for which age groups)?  For which problems (or disorders) have you been able to gain experience with digital interventions?   - *See the list above for suggestions* |
| 6 | What would you say, how do you rate your previous experiences with digital interventions? |
|  | - What did you like and what did you not like? - What was helpful? What was not helpful? - To what extent has the use of digital interventions influenced you or your therapeutic approach? - What made you angry? What bothered you? - Were there things that were negative / positive / disappointing / stimulating / motivating for you? Which? - How did your patients experience the use of digital interventions in treatment? What have your patients told you? |
| 7 | What opportunities and advantages do you see in the care of children and adolescents with eating disorders through digital interventions? |
|  | Do you see specific opportunities and advantages in using a certain technology? If so, which and why?  *🡪 See the list above for suggestions*  Which target groups can benefit from the use of this technology? In what way?   - To what extent could digital offers be helpful in the group of children up to 10 years old with an eating disorder? (Can you describe this group from your professional experience?) - Group of "tweens" (10-13 years) with anorexia - Group of "tweens" (10-13 years) with bulimia - Group of "tweens" (10-13 years) with a binge eating disorder - Group of "teens" (14-19 years) with anorexia - Group of "teens" (14-19 years) with bulimia - Group of "teens" (14-19 years) with a binge eating disorder - Are there other groups that you think should be considered? Which? |
| 8 | What would stop you from using digital interventions in the field of eating disorders for children and adolescents? |
|  | What concerns do you have?  What risks do you see in specific applications? (see list above)  For which target groups do you see critical aspects here?  To what extent do you even see a threat from the use of such applications? |
| 9 | In which sections of care do you think digital interventions for children and adolescents with eating disorders make sense? And why? |
|  | Examples could be:   - Prevention (precaution) - Establish access to the supply system (lower inhibition threshold) - Bridging the waiting time for a therapy place - As a substitute for therapy (= self-help/self-management)   accompanying therapy (in addition to outpatient or inpatient psychotherapy)   - Relapse prevention / aftercare after outpatient or inpatient treatment - Not useful at any time |
| 10 | In which settings could the use of digital interventions for children and adolescents with eating disorders make sense? And why? |
|  | Examples could be:   - Outpatient setting - Inpatient stay - Day clinic - Pure self-management |
| 11 | What requirements must be met so that digital interventions can be meaningfully integrated into the treatment of children and adolescents with eating disorders? |
|  | - To what extent do financial aspects play a role? - Which legal framework conditions are important in your opinion? - What technical requirements must be met? - What requirements would your patients have to meet? - Which further education and training courses would be useful? |
| 12 | I would like to ask you to now imagine that all the prerequisites for the use of digital interventions have been met and that you can support your patients in any way you see fit.  What would an ideal digital intervention for children and adolescents with eating disorders look like?  If possible, please provide one example for anorexia and one for bulimia/binge eating disorder.  (see question 5, asking for specific age groups) |
|  | - What specific aspects of treatment could such an application cover or support? - What technical features would this application offer? - What would be important to you when designing such an application? - On which devices would the application be accessible? - Who would have access to the application? - What roles would parents, relatives or other caregivers play? - What would users have to do to get access to the application? - In what context/ setting would you use the application? - How often should children and adolescents use this program? - What information would be provided to you as a therapist for the treatment? What information would you need? - How would the children and adolescents benefit from this? (What could motivate the children and adolescents to use the intervention?) - To what extent could your therapeutic relationship with the patients (and/or their caregivers) benefit from the application? |
| 13 | Which media do you usually use to obtain information about the treatment of eating disorders in children and adolescents? |
|  | What role play  - Specialist conferences on the topic  - scientific publications on the subject  - the collegial exchange (e.g. in intervision/supervision about current treatment offers)  - professional associations  - Continuing education and training on the subject  for you to find out more about the topic? |
| 14 | Interview conclusion:  Is there anything else that we haven't discussed that you think should be mentioned? Is there anything else you would like to add? |

Thank you very much for your participation!
