# Supplementary material for: Views of German mental health professionals on the use of digital mental health interventions for eating disorders: a qualitative interview study
Source: J Eat Disord. 2024 Feb 23;12:32. doi: 10.1186/s40337-024-00978-1 (PMC10885453; doi:10.1186/s40337-024-00978-1)
Supplement: Supplementary file 2 — Additional file 2. Interview guide for experts in the treatment of adult patients with eating disorders. [file 40337_2024_978_MOESM2_ESM.docx]

# Additional file 2: Interview guide for experts in the treatment of adult patients with eating disorders

General recommendations for using this guide:

- The following guidelines are intended to structure the qualitative interview.
- Both, the order of the questions and their weighting, can be variably adapted to the flow of the conversation over the course of the conversation.
- It's not about repeating the questions exactly, what's important is that the other person understands the question and starts talking.
- Further questions not listed here may also arise from the course of the conversation.

The guide is made up of main questions (gray cells) and possible questions (white cells). The main questions are intended to encourage the other person to talk openly and freely. The questions are asked in addition to generate relevant information that has not yet been mentioned.

In principle, the interview partners should themselves give examples from the area of digital interventions. If there is a need to provide some guidance, interviewers can refer to the list below.

For example, digital interventions may include one or more of the following applications:

- Online advice (email, chat)
- Video conferencing-based systems
- Mobile (smartphone) apps
- Fitness bracelets, wearables
- Therapeutic, browser-based programs, online self-help programs
- Interventions from the area of augmented/virtual reality
- Others (e.g. biofeedback, game consoles, video games)

| 1 | To begin with, can you tell me something about yourself?  How old you are, what your professional background is, and where you are currently based.  How many years have you been treating patients with eating disorders? |
| --- | --- |
|  | - What is your university background; what subject did you study? - Which psychotherapeutic procedures do you use in your work (e.g. psychoanalysis, CT)? What psychotherapeutic procedures have you been trained in (psychoanalysis, KVT)? - In which setting do you work with your patients? (e.g. outpatient/inpatient, group/individual treatments)   *If multiple activities, specialties, work settings, or institutions are reported: Ask about focus, e.g.: "Which of the activities you mentioned do you consider your main activity?", "At which of the institutions you mentioned do you work primarily?"* |
| 2 | What role do digital applications play in your personal everyday life? |
|  | - Which digital applications do you usually use? On which devices do you use them and how often? - What do you use digital applications for? What tasks or functions do these applications perform? - How would you describe your everyday experience with digital applications? What impact does the use of Internet applications have on your personal everyday life? - What advantages or opportunities do you see in your personal use of digital applications? - What disadvantages or risks do you see in your personal use of digital applications? - *See the list above for suggestions* |
| 3 | How would you rate your personal skills in using digital applications? |
|  | - In which areas do you feel confident when using digital applications? - In which areas do you feel insecure when using digital applications? To what extent do you see potential for yourself to learn more about it? - Who or what could support you in learning more about using digital applications? |
| 4 | Could you describe the group of patients with eating disorders that you usually deal with in your daily work? (What are the characteristics of these patients?) |
|  | - What is the age of the patients you work with? - Are they usually women or men? - What is the setting in which you work with the patients? - How do these patients come to you? (Are they referred, recommended, or do they come on their own initiative?) - Do these patients come alone or accompanied? If accompanied, who accompanies them? - What role do the patients' reference persons play in their treatment? For example, how are relatives involved in your work? - Which other persons are important in your work with patients (e.g. social workers)? What role do these people play in treatment? |
| 5 | What experience have you had with digital interventions in your professional practice? |
|  | With which groups of patients have you had this experience in each case?  (e.g. for which age groups)?  For which problems (or disorders) have you gained experience with digital interventions?   - *See the list above for suggestions* |
| 6 | How would you say you rate your experience with digital interventions so far? |
|  | - What did you like and what did you not like? - What was helpful? What wasn't? - In what ways did the use of digital interventions affect you or your therapeutic approach? - What did you feel annoyed about? What bothered you? - Were there things that were negative / positive / disappointing / stimulating / motivating for you? Which ones? - How did your patients* experience the use of digital interventions in treatment? What did your patients report back to you? |
| 7 | What opportunities and benefits do you see in the care of patients with eating disorders through digital interventions? |
|  | Do you see specific opportunities and benefits in using a particular technology? If yes: which ones and why?  *🡪 See the list above for suggestions*  Which target groups can benefit from the use of this technology? In what way?   - Patients with anorexia - Patients with bulimia nervosa - Patients with binge eating disorder - Are there other groups that you think should be considered? Which ones? |
| 8 | What would keep you from using digital interventions for eating disorder patients? |
|  | What concerns do you have?  What risks do you see in specific applications? (see list at the beginning)  For which target groups do you see critical aspects here?  To what extent do you even see a threat from the use of such applications? |
| 9 | At which stages of care do you think digital interventions are useful for patients with eating disorders? And why? |
|  | Examples could be:   - prevention (screening) - Creating access to the care system (lowering inhibition thresholds) - Bridge waiting time for therapy place - As a substitute for therapy (= self-help/self-management) - Accompanying therapy (in addition to outpatient or inpatient psychotherapy) - relapse prevention / aftercare after outpatient or inpatient treatment - not useful at any time |
| 10 | In which settings could the use of digital interventions be useful for patients with eating disorders? And why? |
|  | Examples might include:   - outpatient setting - inpatient setting - day hospital - pure self-management |
| 11 | What conditions would have to be met for digital interventions to be meaningfully integrated into the treatment of patients with eating disorders? |
|  | - To what extent do financial aspects play a role? - What legal framework conditions are important in your eyes? - What are the technical requirements? - Which requirements would have to be met by your patients? - What further training would be useful? |
| 12 | I would like to ask you to imagine now that all the requirements for the use of digital interventions are met and that you can support your patients as you see fit.  What would an ideal digital intervention for patients with eating disorders look like?  If possible, please give an example for anorexia and one for bulimia/binge eating disorder. |
|  | - What specific treatment aspects could such an application cover or support? - What technical functions would this application offer? - What would be important to you in the design of such an application? - On what devices would the application be accessible? - Who would have access to the application? - What roles would family members or other caregivers play? - What would users need to do to access the application? - In what context/setting would you use the application? - How often should patients* use this program? - As a therapist, what information would you be provided with for treatment? What information would you need? - What benefits would patients* receive? (What might motivate patients* to use the intervention?) - How could your therapeutic relationship with patients (and/or their caregivers) benefit from its use? |
| 13 | What media do you typically use to learn about eating disorder treatment? |
|  | What role do  - professional conferences on the subject  - scientific publications on the topic  - collegial exchange (e.g. in intervision/supervision about current treatment offers)  - professional associations  - further education and training on the topic  for you to inform yourself about the topic? |
| 14 | Interview conclusion:  Is there anything else we didn't discuss that you think should be mentioned? Is there anything else you would like to add? |

Thank you very much for your participation!
