# Supplementary material for: Views of German mental health professionals on the use of digital mental health interventions for eating disorders: a qualitative interview study
Source: J Eat Disord. 2024 Feb 23;12:32. doi: 10.1186/s40337-024-00978-1 (PMC10885453; doi:10.1186/s40337-024-00978-1)
Supplement: Supplementary file 3 — Additional file 3. Summary of the main codes in alphabetic order used for qualitative analysis with their subcodes and supporting quotes. [file 40337_2024_978_MOESM3_ESM.docx]

**Additional file 3: Summary of the main codes in alphabetic order used for qualitative analysis with their subcodes and supporting quotes**

| **Codes** | **Definition** | **Subcodes** | **2^nd^ level Subcodes** | **Supporting quotes** |
| --- | --- | --- | --- | --- |
| Initial situation of the interviewed person | Expressions referring to the professional background of the interview partner | working conditions, treatment setting |  | *… we actually do interval therapies, which means that the anorexia patients in particular are treated inpatient with us for a few weeks, then return to their home environment and are then admitted again and then return to their home environment and are again admitted, as many intervals as are somehow necessary*. (psychological psychotherapist in training, adult patients, full-time inpatient care, female, 26 years) |
|  |  | role of companions and other experts |  | *In general, we always try to have a discussion with relatives or couples, because it is actually very common that relatives are involved in the eating disorder.* (psychological psychotherapist in training, adult patients, full-time inpatient care, female, 26 years) |
|  |  | sources of information |  | *As part of the training, we had seminars on the treatment of eating disorders. As part of this training for the study, there were also a few online events on the topic and clearly team meetings and collegial exchange also play a role.* (psychological psychotherapist, adult patients, outpatient care, female, 32 years) |
|  |  | patients' characteristics |  | *In principle, we treat all common forms of eating disorders, i.e. anorexia, bulimia, binge eating and the intermediate forms. Here, however, the distribution is such that about two thirds are anorexia patients, one third bulimia and only in individual cases binge eating patients.* (psychological psychotherapist, adult patients/children and adolescent patients, part-time inpatient care, male, 31 years) |
|  |  | use of media in general | general; private; professional | *My personal competence or experience was pretty low before Corona and then we somehow had to switch to offering video calls here in the outpatient clinic quite suddenly. And that actually worked quite well relatively quickly*. (Specialist in psychiatry, psychotherapy and/or psychosomatics, adult patients, outpatient, female, 58 years) |
|  |  | provision of care |  | *Most of them come, so we always do a pre-inpatient consultation. They are referred to us either by the pediatrician or the family doctor, or very often by established psychotherapists or adolescent psychotherapists.* (Specialist in psychotherapy and psychosomatics, children and adolescent patients, full-time /part-time inpatient care, male, 55 years) |
| General attitudes towards digital interventions | Expressions of general opinions and attitudes toward digital developments in health care | curiosity, interest |  | *- What would you say, how do you rate your personal skills in dealing with digital applications?* (Interviewer) *- Well I'm quite open to experimentation, so I don't have any reservations about that.* (Specialist in internal medicine and cardiology, additional psychotherapist/senior physician, adult patients, inpatient/part-time inpatient care, female, 43 years) |
|  |  | skepticism, insecurity |  | *And that's why I have a bit of doubt that you can really do that with digital media, that you can take a preventive approach.* (Specialist in internal medicine and emergency medicine, further training in psychosomatic medicine and psychotherapy, adult patients, inpatient/part-time inpatient care, female, 37 years) |
|  |  | future prospects |  | *Well, we are also doing something in the direction of digitization and also digital interventions, so I think we, yes, our clinics, yes, so to speak, yes, there too, I think, will also be involved in such development processes in the future.* (Specialist in internal medicine and cardiology, additional psychotherapist/senior physician, adult patients, inpatient/part-time inpatient care, female, 43 years ) |
| General societal conditions | Expressions referring to trends in healthcare or in society not specific for digital interventions | care supply in general |  | *We simply have such unbelievable difficulties in accommodating people. There are so few therapists specialized in eating disorders that I am very ... very annoyed about the fact that since 1998 we have had to have this discussion about health insurance.* (psychological psychotherapist, adult patients, full -time inpatient care, female, 55 years). |
|  |  | social media |  | *And also, overall on social media, Instagram or so, it is of course a topic with the wards, when the patients post any pictures of themselves, how thin their belly is, or how muscular their six pack is.* (Specialist in psychotherapy and psychosomatics, children and adolescent patients, full-time/part-time inpatient care, male, 55 years ) |
|  |  | COVID-19 pandemic |  | *In this respect, this Corona time was helpful in order to see what works. what is good and what is not so good and what is useful and what is not useful.* (psychological psychotherapist, adult patients, full-time inpatient care, female, 55 years ) |
| Experiences with digital interventions | Expressions reporting on own experiences made by the interview partners regarding digital interventions for mental health care | own developments or evaluations |  | *Otherwise, I was involved in study B, there we were comparing face to face cognitive behavioral therapy vs. guided self-help. That is my experience with patients with binge eating disorder in the digital domain. I was project leader in place J.* (psychological psychotherapist and scientific activity, adult patients/children and adolescent patients, outpatient care, female, 47 years) |
|  |  | impact on therapeutic process |  | *As I've noticed or experienced, when I offer therapy online I'm maybe not as confrontational sometimes or maybe can't go as deep into the topic because of course I can't respond immediately.* (psychological psychotherapist, children and adolescent patients, outpatient care, female, 47 years) |
|  |  | specific functionalities |  | *So, what I like to recommend, which I have also recommended a lot in psychosomatics, is relaxation apps. This is more widely spread, but I like to recommend it, because it gives easy access to relaxation techniques.* (Specialist in child and adolescent psychiatry and psychotherapy, children and adolescent patients, outpatient care, female, 38 years) |
|  |  | media |  | *So, until now, I've actually only ever used video conferencing systems or just this app for the meal logs and I don't think I've used anything else.* (psychological psychotherapist in training, adult patients, full -time inpatient care, female, 26 years ) |
|  |  | patients' characteristics |  | *So, I would say that it is more centered on adolescents than it is on younger adults and adolescents, ... who also inform themselves very broadly and that this is really a source for information and a source for help.* (psychological psychotherapist, adult patients, part-time inpatient care, female, 31 years) |
|  |  | experiences made by patients |  | *As a rule, I have received feedback where they give it a positive evaluation because it is relatively close to their everyday life and the hurdles are then also present and apparently also a bit lower.* (psychological psychotherapist, children and adolescent patients, full-time inpatient care, female, 41 years) |
|  |  | setting |  | *Well, I have become acquainted with various interventions within the framework of studies and I have also made experiences. I myself have conducted follow-up groups in the online format.* (psychological psychotherapist, adult patients, outpatient care, female, 40 years) |
| Advantages and chances | Expressions describing advantages and chances of the use of digital interventions for eating disorders | advantages for relatives |  | *And as far as the relatives are concerned, yes, perhaps it would relieve them to some extent if, that is, if it were now a matter of this compliance with, with structure. Otherwise, If that is always a friendly reminder from the wife then that just ceases to exist because it now happens digitally via an app, a reminder, exactly, that could perhaps relieve the relationship.* (specialist in internal medicine and cardiology, additional psychotherapist/senior physician, adult patients, inpatient/part-time inpatient care, female, 43 years) |
|  |  | advantages for care supply | possibility to control adherence;  cost-effectiveness;  therapeutic alliance; quality of care | *It could just as well help as a control instrument if the patient's statements aren't really conclusive, but, I know, I don't know specifically whether that's the case now.* (specialist in internal medicine and cardiology, additional psychotherapist/senior physician, adult patients, inpatient/part-time inpatient care, female, 43 years) |
|  |  | advantages for patients | proximity to every day conditions, low-threshold, flexibility;  autonomy, self-determination;  enrichment, motivation, commitment;  interactivity;  privacy, anonymity;  psycho-education;  reduction of shame; self-reflection;  stabilising, structuring;  linkage | *I really believe that the biggest advantage is that the inhibition threshold is lower. Many people have an incredibly big problem with their bodies, with showing themselves, with shame, with all of that. Consequently, such a personal contact is incredibly challenging for many anyway. And I believe that because this is no longer the case with such a medium, I think you can reach many more people who do not yet dare to do so.* (psychological psychotherapist, adult patients/children and adolescent patients, part-time inpatient care, male, 31 years) |
| Disadvantages and boundaries | Expressions describing disadvantages, boundaries or risks of the use of digital interventions for eating disorders | disadvantages for care supply | loss of therapeutic alliance; lacking information and expertise; lacking control; costs, resources; legal consequences;  technical issues;  replacement for therapy; feeling disturbed | I *think I would find it unfavorable if it only came down to doing something digitally, because you can't see yourself and I don't want that to sound so negative, but I think you can hide yourself well on the screen. So I get more from my patients, emotionally and of course also visually when I have them sitting in front of me, especially with patients with an eating disorder*. (Specialist in internal medicine and emergency medicine, further training in psychosomatic medicine and psychotherapy, adult patients, inpatient/part-time inpatient care, female, 37 years) |
|  |  | disadvantages for patients | lacking privacy;  lacking individualization;  lacking private sphere, lacking quietness; insufficient support;  increasing media consumption;  worsening of illness | *I think it's generally the case with eating disorder patients that it's important that they learn to accept help, and often one of the crucial points is always: I have to manage this on my own, I have to have managed this on my own, and that's part of the disease. And if they then manifest that through digital offers, where you just don't have to accept that help as well, then I think that's part of the disease itself just not processed.* (psychological psychotherapist, children and adolescent patients, outpatient care, female, 47 years) |
| Desired functions and properties | Expressions referring to single functions and properties in an ideal digital intervention as imagined by the interview partners | design | usability; co-creation; patients' perspective; layout; reminders,  personalization;  gamification, interactivity | *So, I think I would think it would be cool if you could, I imagine if you could click the scale up or down or that you could have like a laughing smily or something, I think the more you can swipe and press, the easier it is, the less it feels like work.* (specialist in child and adolescent psychiatry and psychotherapy, children and adolescent patients, outpatient care, female, 38 years) |
|  |  | media | mobile app; biofeedback  e-mail; wearable device; online-platform;  videoconference, camera; virtual reality | *So, in fact, ideally, I would prefer a hybrid model, but that would be that the first contact takes place live with the anorexia patient and then at the same time in this first contact the patient can be given an app with which she can inform herself in parallel about her eating disorder and the treatment of the eating disorder.* (psychological psychotherapist, adult patients, outpatient care, female, 40 years ) |
|  |  | technical functionalities | content regulations, storage; communication, contact functionalities (for relatives; for therapists; for patients; common access); compatibility | *That would be a bit like a diary ... So, I don't think that parents or therapists should or would be allowed to look into it, but that it would be included in the therapy so to speak, but that the patient then decides for themselves what or how much they want to show. As I said, similar to a diary or an eating protocol.* (specialist in psychotherapy and psychosomatics, children and adolescent patients, full-time/part-time inpatient care, male, 55 years) |
|  |  | therapeutic content | awareness, relaxation  sports  diagnosis, screening; exposition, confrontation;  crisis intervention;  motivation, affirmation;  psycho-education;  resources, skills;  structuring, eating plans; exercises, home work; progress monitoring; protocols | *Yes, perhaps also an area where it would be possible to learn some skills, i.e. some techniques, strategies for difficult emotions or for states of tension. This is often something that triggers the eating disorder, and these are things that can be learned quite easily at a very low level.* (psychological psychotherapist, adult patients/children and adolescent patients, part-time inpatient care, male, 31 years)  *And then I would, yes, I think I would put it on different pillars, where there is a part where there is a lot of psychoeducation, simply learning a lot about the disease and about yourself, a few questions, then a part where it is perhaps about nutrition, where there are also exemplary meal plans or where you can learn a bit playfully, which portion sizes are normal, which are not.* (psychological psychotherapist, adult patients/children and adolescent patients, part-time inpatient care, male, 31 years) |
| Target groups of digital interventions | Expressions referring to potential target groups and their properties of digital interventions for eating disorders | patients’ age |  | *Yes, so spontaneously I would think that the young patients probably benefit better, so starting from 14 and 15 I can imagine there is more personal responsibility and, perhaps also more ability to reflect required than if you work something out with direct assistance in a conversation with a therapist.* (psychological psychotherapist, children and adolescent patients, full-time inpatient care, female, 41 years) |
|  |  | gender |  | *Maybe also young men, so that's similar, that that's also often still difficult for them to show themselves or to say, yes, I'm a boy, I'm a young man and I have an eating disorder.* (psychological psychotherapist, adult patients/children and adolescent patients, part-time inpatient care, male, 31 years) |
|  |  | patients' motivation, initiative, interest |  | *So, with the eating protocol, I would think that personal responsibility would have to be that they do that without being asked and without being constantly reminded.* (psychological psychotherapist, adult patients, full-time inpatient care, female, 55 years) |
|  |  | media competency |  | *Yes, so I think it has to be clarified in advance: Can they handle it? Especially if it is app-based. It's no use if I try to implement it, but patients don't know at all how I can manage it.* (psychological psychotherapist, adult patients, part-time inpatient care, female, 31 years) |
|  |  | diagnosis | adiposis; alcohol use disorder;  anxiety; anorexia;  binge eating disorder;  bulimia;  depression;  eating disorders (general);  psychosocial functioning;  comorbidities;  illness severity | *Of course, it also depends on the clinical picture. It is possible, one could imagine that one develops something that has psychoeducational content so that people realize: Okay, I have an eating disorder. I mean, patients with anorexia usually know that very well, and those with bulimia also.* (specialist in internal medicine and cardiology, additional psychotherapist/senior physician, adult patients, inpatient/part-time inpatient care, female, 43 years) |
|  |  | contra-indication | general;  mobbing, trauma;  gaming addiction, internet addiction;  suicidality, self-harm | *Well, there is also the risk of online addiction or online gaming or whatever. There is then, such a dichotomy that on the one hand, if people are very active online and have an interest then of course you can pick them up easy, but that on the other hand, of course, you also strengthen their online attachment again, which I would find problematic.* (psychological psychotherapist, adult patients/children and adolescent patients, part-time inpatient care, female, 51 years) |
|  |  | social environment, relatives |  | *So, with digital media use with younger children, of course parents would have to get on board. But I can't think of anything right now, that has much to do with eating disorders.* (child and adolescent psychotherapist, children and adolescent patients, outpatient care, female, 45 years) |
| General conditions and requirements |  | spacial conditions |  | *And it has to be, so when it comes to online therapies it needs to be a safe space, which is not so easy and self-evident.* (specialist in psychosomatics in training, adult patients, outpatient care, female, 40 years) |
|  |  | scientific evidence |  | *And that it also evaluates a part, I would also say that that is also important, look a bit, does it bring something? Does it have a certain effect?* (psychological psychotherapist, adult patients, full-time/part-time inpatient care, female, 55 years) |
|  |  | costs, finances |  | *So that's the question, whether more effort is required, and if so, it would of course be good if that were somehow rewarded.* (psychological psychotherapist, adult patients/children and adolescent patients, part-time inpatient care, male, 31 years) |
|  |  | vocational training |  | *I think, so I would definitely like to have training on exactly how that works....* (psychological psychotherapist in training, adult patients, full-time inpatient care, female, 26 years) |
|  |  | indication |  | *I would like to know for which patient group this is recommended and why.* (psychological psychotherapist, adult patients, outpatient care, female, 31 years) |
|  |  | health insurance |  | So, I think it would be good if when that comes on the market and it is planned, that it is somehow used more widely if the health insurance company takes it over. (psychological psychotherapist in training, adult patients, full-time inpatient care, female, 26 years) |
|  |  | frequency and degree of use |  | *So, when it comes to logging, it would be useful if it could be exactly daily and at least once a week to be able to view or upload things again.* (psychological psychotherapist, children and adolescent patients, part-time inpatient care, female, 33 years) |
|  |  | availability of offers |  | *I think the lack of availability keeps me from doing that. If we had that, if we could offer that, I think I would try that out to see how it goes.* (specialist in internal medicine and emergency medicine, further training in psychosomatic medicine and psychotherapy, adult patients, inpatient/part-time inpatient care, female, 37 years) |
|  |  | staff prerequisites |  | *And then, of course, everyone involved has to feel comfortable with it. So when I think about our team here now, I think there would also be headwind from one or more of the long-established people.* (specialist in internal medicine and emergency medicine, further training in psychosomatic medicine and psychotherapy, adult patients, inpatient/part-time inpatient care, female, 37 years) |
|  |  | legal conditions |  | *And legally, of course, there is always the question of data protection, i.e., what happens to the data that people enter, for example, in an online course or in an app, how is it protected, who regulates it, and that should also be included as information in principle.* (psychological psychotherapist, adult patients, outpatient care, female, 31 years ) |
|  |  | therapeutic setting | consulting; group setting; care supply in general;  transitions of care;  after-care, relapse prevention;  prevention, early detection; school;  self-management;  treatment-related (in general; outpatient; inpatient; day-care); bridging waiting time | *So ultimately, I think you can use that, that is, if the offers are there, then you can use them, not only in one setting. So, I mean, I think that's exactly the point, that you say: Okay, no matter whether a patient is cared for as an outpatient or as an inpatient or as a day patient or whatever, you can use your digital interventions.* (psychological psychotherapist, adult patients, part-time inpatient care, female, 31 years) |
|  |  | technical requirements |  | *Yes, they must somehow now have a PC and or a smartphone, at least for the two things that I have tried out so far. In this sense, that is already a prerequisite that somehow everyone has been able to fulfill up to now.* (psychological psychotherapist in training, adult patients, full-time inpatient care, female, 26 years) |
|  |  | therapeutic alliance as a basis |  | *But basically, I don't think I would want to run it exclusively through that. I still use this eating protocol app with a patient I've known for a long time with whom I have a good relationship, and it works great. But now I only do it with this one patient for reasons I mentioned before.* (Specialist in internal medicine and emergency medicine, further training in psychosomatic medicine and psychotherapy, adult patients, inpatient/part-time inpatient care, female, 37 years) |
